# Repetitive DNAs and shrink genomes: A chromosomal analysis in nine Columbidae species (Aves, Columbiformes)

**DOI:** 10.1590/1678-4685-GMB-2017-0048

**Published:** 2018-02-19

**Authors:** Rafael Kretschmer, Thays Duarte de Oliveira, Ivanete de Oliveira Furo, Fabio Augusto Oliveira Silva, Ricardo José Gunski, Analía del Valle Garnero, Marcelo de Bello Cioffi, Edivaldo Herculano Corrêa de Oliveira, Thales Renato Ochotorena de Freitas

**Affiliations:** 1 Programa de Pós-Graduação em Genética e Biologia Molecular, PPGBM, Universidade Federal do Rio Grande do Sul, Porto Alegre, Rio Grande do Sul, RS, Brazil; 2 Programa de Pós-Graduação em Ciências Biológicas, PPGCB, Universidade Federal do Pampa, São Gabriel, Rio Grande do Sul, RS, Brazil; 3 Programa de Pós-Graduação em Genética e Biologia Molecular, PPGBM, Universidade Federal do Pará, Belém, PA, Brazil; 4 Instituto de Ciências Exatas e Naturais, Universidade Federal do Pará, Belém, PA, Brazil; 5 Departamento de Genética e Evolução, Universidade Federal de São Carlos, São Carlos, SP, Brazil; Laboratório de Cultura de Tecidos e Citogenética, SAMAM, Instituto Evandro Chagas, Ananindeua, PA, Brazil

**Keywords:** Birds, FISH, microsatellites, sex chromosomes, chromosomal rearrangements

## Abstract

An extensive karyotype variation is found among species belonging to the Columbidae family of birds (Columbiformes), both in diploid number and chromosomal morphology. Although clusters of repetitive DNA sequences play an important role in chromosomal instability, and therefore in chromosomal rearrangements, little is known about their distribution and amount in avian genomes. The aim of this study was to analyze the distribution of 11 distinct microsatellite sequences, as well as clusters of 18S rDNA, in nine different Columbidae species, correlating their distribution with the occurrence of chromosomal rearrangements. We found 2n values ranging from 76 to 86 and nine out of 11 microsatellite sequences showed distinct hybridization signals among the analyzed species. The accumulation of microsatellite repeats was found preferentially in the centromeric region of macro and microchromosomes, and in the W chromosome. Additionally, pair 2 showed the accumulation of several microsatellites in different combinations and locations in the distinct species, suggesting the occurrence of intrachromosomal rearrangements, as well as a possible fission of this pair in *Geotrygon* species. Therefore, although birds have a smaller amount of repetitive sequences when compared to other Tetrapoda, these seem to play an important role in the karyotype evolution of these species.

## Introduction

Columbiformes is one of the most easily recognized bird orders in the world, with more than 300 species and traditionally divided into two families: Columbidae (pigeons and doves) and Raphidae (Pereira *et al.*, 2007). Three large clades are supported on Columbiformes, referred to as A, B, and C by Pereira *et al.* (2007), based on mitochondrial and nuclear DNA data. Clade A is subdivided into two well-supported subclasses: one referring exclusively to America genera and the other includes pigeons and turtle doves from the Old and New Worlds. Clade B groups only New World pigeon species and Clade C includes many genera found in Africa, Asia, Australia, the East Indies, and New Zealand.

Cytogenetic studies based mainly on conventional staining have shown an interesting variation in diploid number, which ranges from 76 to 86 (Takagi and Sasaki, 1974; de Lucca and de Aguiar, 1976; de Lucca, 1984). Other aspects of their karyotypical organization remain unknown, although the observed variation in chromosome morphology suggests the occurrence of intra- and interchromosomal rearrangements (de Lucca, 1984).

There is evidence supporting that some groups of vertebrates with a high metabolic demand have smaller cells, and as consequence, smaller genomes (Szarski, 1983). In accordance with this hypothesis, the relationship between flying and the reduced genome size of birds, bats and possibly pterosaurs, has been interpreted as an evidence that the high energetic demand of flying exerted selective pressures for small cells and small genomes (Hughes and Hughes, 1995; Organ and Shedlock, 2009; Zhang and Edwards, 2012). Conformingly, birds have the lowest average genome sizes among Tetrapoda (Andrews *et al.*, 2009) while bats show the smallest genomes when compared to most Mammalian species (Smith and Gregory, 2009). In addition, humming birds have the smallest genomes among birds, probably associated with their intense necessity of energy to hover during flight (Gregory *et al.*, 2009).

Repetitive DNAs represent an important proportion of the genome in eukaryotes, being composed by sequences *in tandem* (satellites, minisatellites and microsatellites) and transposable elements (transposons and retrotransposons) (Charlesworth *et al.*, 1994; López-Flores and Garrido-Ramos, 2012). These repetitive sequences play an important role in genome evolution in eukaryotes (Biémont and Vieira, 2006). For example, it was proposed that the genome evolution in mammals has been driven by chromosomal rearrangements in fragile sites, composed by *in tandem* repetitive sequences (Ruiz-Herrera *et al.*, 2006). In addition, transposable elements can also influence the occurrence of chromosomal rearrangements by inducing chromosomal breakage (Biémont and Vieira, 2006).

An important class of repetitive sequences is formed by the microsatellites, small sequences (1–6 base pairs) repeated *in tandem* and dispersed through the genome. Mono-, di-, tri-, and tetranucleotide repetitions are the most common types of microsatellites (Ellegren, 2004). Mutation rates in these sequences are 10-100,000 folds higher than the mean of other genome regions, making them important markers for genetic variability studies of natural and captive populations (Gemayel *et al.*, 2010). Cytogenetic mapping of these sequences has also contributed to a better comprehension of sex chromosome evolution and chromosomal differentiation, and have been extensively analyzed in fishes (Cioffi and Bertollo, 2012). In general, repetitive sequences accumulate preferentially in centromeric and heterochromatic regions, as observed in many fishes (Cioffi *et al.*, 2012), lizards (Pokorná *et al.*, 2011) and plant species (Kejnovsky *et al.*, 2013). However, little is known about the dynamic of repetitive sequences in birds. In sauropsids (reptiles and birds), many microsatellites have been intensely amplified in sex chromosomes Y/W in seven species (six reptiles and *Gallus gallus*), associated to the differentiation and heterochromatinization of these chromosomes (Matsubara *et al.*, 2015).

Recently, distinct hybridization patterns of microsatellite sequences have been demonstrated in species of two different orders of birds (de Oliveira *et al.*, 2017; Furo *et al.*, 2017). In Piciformes, a large accumulation of 10 sequences was observed on autosomes and especially on the Z sex chromosome in three woodpecker species (Picidae). The Z chromosome corresponds to the larger element of their karyotype due to the accumulation of such sequences, which increased its size (de Oliveira *et al.*, 2017). On the other hand, in *Myiopsitta monachus* (Psittaciformes, Psittacidae) these sequences accumulated preferentially in the W sex chromosome, which has the same size of the Z chromosome, unlike most Neognathae bird species (Furo *et al.*, 2017). These two examples show that the analysis and mapping of repetitive sequences in the genome of avian species may contribute for a better understanding of the processes underlying sex chromosomes differentiation and karyotype evolution.

Thus, the analysis of microsatellite sequences in groups of birds showing chromosomal variation both in diploid number and chromosomal morphology, such as Columbiformes, may bring important information concerning their karyotypical evolution. In this study, we report the chromosomal mapping of different repetitive sequences, including 18S rDNA clusters and 11 different microsatellite sequences in Columbidae species in order to verify the role of these sequences in their karyotypical diversity. The results suggest that, despite their lower amount in the genome, repetitive DNAs seem to play an important role in the karyotype evolution of these species.

## Material and Methods

### Specimens and chromosome preparations

Nine species of Columbidae family were analyzed in this study. Individuals were collected in their natural habitat, except for *G*. *montana* and *G*. *violacea*, which were collected from captivity (Table 1). Experiments followed protocols approved by the Ethics Committee on the Use of Animals (CEUA - Universidade Federal do Pampa, 026/2012, and permission number SISBIO 33860-1 and 44173-1).

**Table 1:** Information concerning the individual samples used for this study.

Species	Number of individuals/Sex	City/State^*^
*Zenaida auriculata*	2 M	São Gabriel/RS
*Leptotila verreauxi*	1 M and 2 F	Santa Maria/RS
*Columba livia*	1 F	São Gabriel/RS
*Columbina picui*	2 M	Santa Maria and Porto Vera Cruz/RS
*Columbina passerina*	1 M	Belém/PA
*Columbina talpacoti*	3 M and 1 F	Porto Vera Cruz/RS
*Patagioenas cayennensis*	2 M	Porto Vera Cruz /RS
*Geotrygon violacea*	1 F	Belém/PA
*Geotrygon montana*	1 M	Belém/PA

^*^ Brazilian States: RS, Rio Grande do Sul; PA, Pará. M = Male. F = Female.

Chromosomes were obtained from fibroblast cultures, according to Sasaki *et al.* (1968) or from bone marrow, following Garnero and Gunski (2000). Both techniques included exposition to colcemid (1 h, 37 ºC), hypotonic treatment (0.075 M KCl, 15 min, 37 ºC) and fixation with methanol/acetic acid (3:1).

### Chromosome probes and FISH experiments

18S rDNA fragments were amplified by PCR using primers NS1 5’-GTA GTC ATA TGC TTG TCT C-3’ and NS8 5’-TCC GCA GGT TCA CCT ACG GA-3’ and nuclear DNA of *Ocyurus chrysurus* (Perciformes: Lutjanidae) (White *et al.*, 1990). Subsequently, fragments were labeled with digoxigenin by nick translation (Roche) and detected with anti-digoxigenin-rhodamine, following the manufacturer’s instructions. Preparation of slides, hybridization and washes were performed according to Daniels and Delany (2003).

FISH experiments using microsatellite probes were done according to Kubat *et al.* (2008). Oligonucleotides (CA)_15_, (CAA)_10_, (CAC)_10_, (CAG)_10_, (CAT)_10_, (CG)_15_, (CGG)_10_, (GA)_15_, (GAA)_10_, (GAG)_10_ and (TA)_15_, directly labeled with Cy3 at the 5terminal were obtained from SIGMA. After denaturation, probes were applied on the slides and incubated for 16 h at 37 ºC in a humid chamber. Next, slides were washed twice in 2xSSC, twice in 1xSSC, and in PBS (phosphate buffered saline), and then dehydrated in an ascending ethanol series (70, 90 and 100%).

At least 30 metaphase spreads were analyzed to confirm the 2n, karyotype structure and FISH results. Images were captured using a Zeiss Imager Z2, coupled with the software Axiovison 4.8 (Zeiss, Germany). The chromosomes were classified as metacentric (m), submetacentric (sm), telocentric (t) or acrocentric (a) according to their arm ratios (Guerra, 1986).

## Results

**Figure 1:**
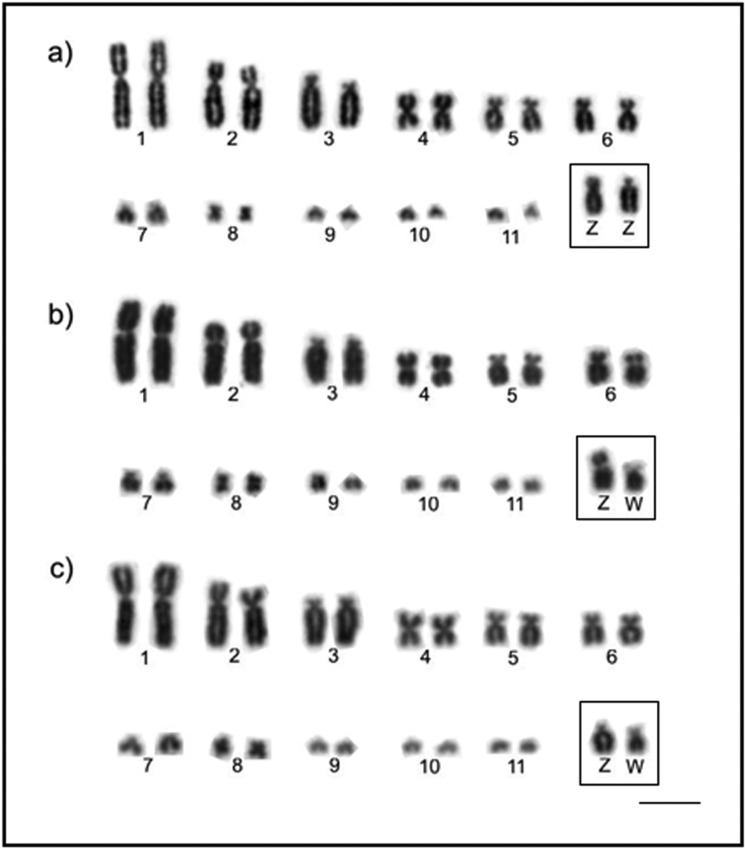
Partial karyotype showing the largest autosomal pairs and ZW sex chromosomes of three *Leptotila verreauxi* individuals analyzed by conventional Giemsa-staining: (a) male with a submetacentric and acrocentric Z chromosomes; (b) female with submetacentric Z and W chromosomes, (c) female with an acrocentric Z and a submetacentric W chromosome. Sex chromosomes are boxed. Bar = 5 μm.

**Figure 2:**
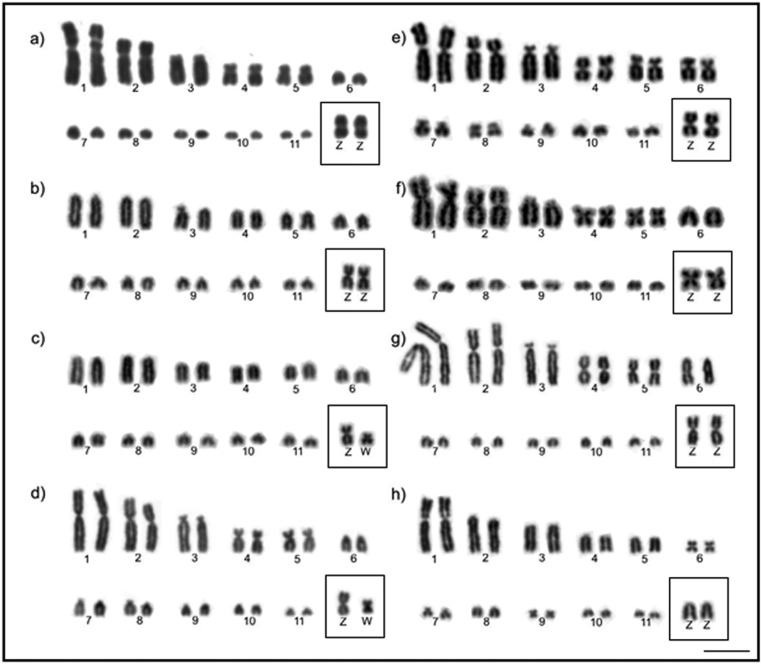
Partial karyotype showing the largest autosomal pairs and ZW sex chromosomes of eight Columbidae analyzed by conventional Giemsa-staining: (a) *Zenaida auriculata*, male; (b) *Geotrygon montana*, male; (c) *Geotrygon violacea*, female; (d)*, Columba livia*, female; (e) *Patagioenas cayennensis*, male; (f) *Columbina talpacoti*, female; (g) *Columbina passerina*, male; (h) *Columbina picui*, male. Sex chromosomes are boxed. Bar = 5 μm.

Diploid number and chromosomal morphology of the species analyzed are described in Table 2. Figures 1 and 2 show the karyotypes in conventional staining. We found a morphological variation in the Z chromosome of *L*. *verreauxi*, which corresponded to a submetacentric or acrocentric element (Figure 1). Additionally, pair 3 also showed morphological variation in *G*. *montana* as telocentric and acrocentric (Figure 2b).

**Table 2:** Diploid number and chromosomal morphology of the nine Columbidae species included in this study.

	Chromosomes												
Species	2n	1	2	3	4	5	6	7	8	9	10	Z	W
*Z. auriculata*	76	SM	SM	A	SM	SM	T	T	T	T	T	M	-
*G. montana*	86	T	T	*	T	T	T	T	T	T	T	M	-
*G. violacea*	86	T	T	T	T	T	T	T	T	T	T	M	SM
*L. verreauxi*	78	SM	SM	A	M	A	A	A	M	T	T	*	SM
*C. livia*	80	SM	SM	A	SM	SM	T	T	T	T	T	M	M
*P. cayennensis*	76	SM	SM	A	M	A	A	A	T	T	T	M	-
*C. talpacoti*	76	SM	SM	A	M	M	T	T	T	T	T	M	-
*C. passerina*	76	SM	SM	A	M	M	T	T	T	T	T	M	-
*C. picui*	76	SM	T	T	T	T	M	A	T	M	T	T	-

2n = diploid number, M = metacentric, SM = submetacentric, A = acrocentric, T = telocentric, * = variable morphology.

**Figure 3:**
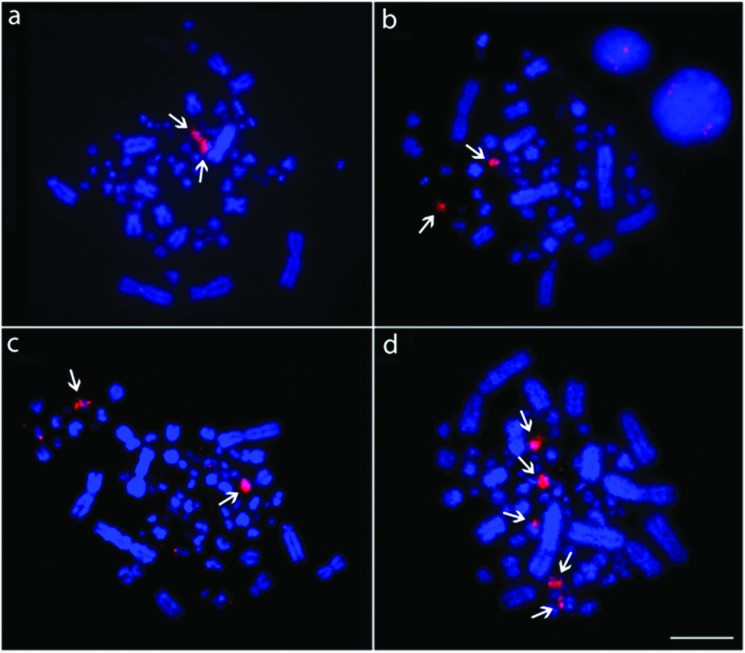
Representative examples of FISH experiments using 18S rDNA probes in Columbidae species. (a) *L*. *verreauxi*; (b) *Z*. *auriculata*; (c) *C*. *livia*; (d) *C*. *picui*. The arrows point to the hybridization signals. Bar = 5 μm.

18S rDNA probes hybridized onto microchromosomes in the nine species analyzed here. In *Z*. *auriculata*, *G*. *montana*, *G*. *violacea*, *L*. *verreauxi*, *P*. *cayennensis*, *C*. *livia*, *C*. *talpacoti* and *C*. *passerina* this sequences were detected in only one microchromosome pair, however, in *C*. *picui* these probes revealed the presence of clusters in three pairs of microchromosomes. Examples of 18S rDNA hybridization in the Columbidae are shown in Figure 3.

### Chromosome mapping of microsatellite sequences

**Figure 4:**
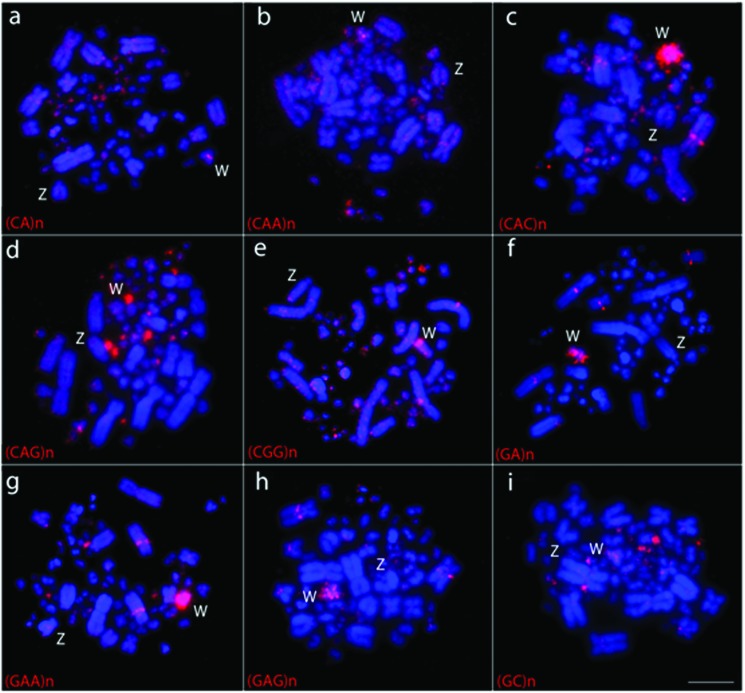
Metaphases of a female *Leptotila verreauxi* in experiments of FISH using nine different microsatellite sequences (a-i). Chromosomes were counterstained with DAPI (blue) and probes were directly Cy3 (red) labeled. Microsatellite sequences are indicated on the bottom left of each figure. Sex chromosomes are indicated in each metaphase. Bar = 5 μm.

**Figure 5:**
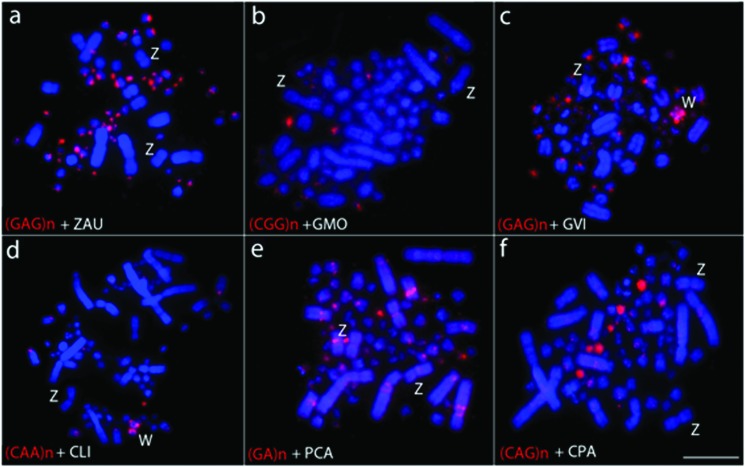
Representative examples of FISH experiments using microsatellite sequences in six Columbidae species (a-f). Probes were directly labeled with Cy3 (red), while chromosomes were counterstained with DAPI (blue). Microsatellite sequences are indicated on the bottom left of each figure. Sex chromosomes are indicated in each metaphase. ZAU: *Zenaida auriculata* (a); GMO: *Geotrygon montana* (b); GVI: *Geotrygon violacea* (c); CLI: *Columba livia* (d); PCA: *Patagioenas cayennensis* (e); CPA: *Columbina passerina* (f). Sex chromosomes are indicated in each metaphase. Bar = 5 μm.

Of the nine species analyzed, only *C. picui* showed no hybridization signals for the microsatellite sequences used. In this species, we performed the hybridizations with chromosomal preparations obtained from two distinct protocols, fibroblasts and direct culture of bone marrow and obtained the same negative result. The other species showed an exclusive pattern of distribution for at least some of the microsatellite sequences used (Table 3). In general, these sequences were preferentially accumulated in the centromeric region of some macrochromosome pairs, in microchromosomes and in the W chromosome. There was no evident signal in the Z chromosome of any species. In addition, pair 2 showed an interesting accumulation of some sequences, of which the position varied in some species – a single band in the short arms in *Z*. *auriculata*, *C*. *passerina* and *C*. *talpacoti*, a single band in the long arms in *L*. *verreauxi*, *G*. *montana* and *P*. *cayennensis*, and two bands (GA_15_) in the short arms in *P*. *cayennensis*. The highest number of sequences was found in *L*. *verreauxi* (Figure 4). Representative experiments of other species are shown in Figure 5.

**Table 3:** Hybridization of 11 microsatellite sequences in nine Columbidae species.

Repeat motif	Species								
	ZAU	LVE	PCA	GVI	GMO	CLI	CPI	CTA	CPA
(CA)_15_	Centromere of machrocromosomes	Pericentromeric region of 2p; W centromere	Pericentromeric region of pairs 5 and 6; 2q	-	-	Centromere pairs 6-10	-	Pericentromeric region of 2p; telomere of 2p and 1p; centromere of pair 5	Pericentromeric region of 2p; telomere of 1p; centromere of pair 4; one pair of microchromosome
(TA)_15_	-	-	-	-	-	-	-	-	-
(GA)_15_	Most microchromosomes	Pericentromeric region of 2p; W q and p; centromere of pair 5	Two blocks in 2q	Chromosome W p and q; 2p	2q; 4q	Two pairs microchromosomes; all chromosome W	-	Pericentromeric region of 2p	2p
(CAA)_10_	-	W centromere	-	Some microchromosomes	Two pairs of microchromosomes	Centromere pairs 6-10; all chromosome W	-	-	-
(GAA)_10_	Pericentromeric region of 2p; centromere of most microchromosomes	Pericentromeric region of 2p; Wq; one pair of microchromosomes	2q	-	-	-	-	Pericentromeric region of 2p; centromere of pair 4; telomere of 1q	2p
(CAT)_10_	-	-	-	-	-	-	-	-	-
(GC)_15_	-	Some microchromosomes	-	-	-	-	-	-	-
(CGG)_10_	One pair of microchromosomes	Pericentromeric region of 2p; terminal region of W	One pair of microchromosomes	-	Two pairs of microchromosomes	Two pairs of microchromosomes; all chromosome W	-	One pair of microchromosomes	-
(CAG)_10_	-	Some microchromosomes	Three pairs of microchromosomes	-	-	-	-	One pair of microchromosomes; centromere of 6 pair	Some microchromosomes; centromere of pair 6
(CAC)_10_	-	Pericentromeric region of 2p; W centromere and q	Pericentromeric region of pair 5	-	-	-	-	Pericentromeric region of 2p; telomere of 1p	2p
(GAG)_10_	Most microchromosomes	Pericentromeric region of 2p; Wq	Some microchromosomes; 2q	Some microchromosomes	Some microchromosomes	Some microchromosomes; all chromosome W	-	Telomere and centromere of pair 6; Some microchromosomes	Some microchromosomes; centromere of pair 6

(-) no hybridization signals.

## Discussion

Corroborating previous studies (Takagi and Sasaki, 1974; de Lucca and de Aguiar, 1976; de Lucca, 1984) we observed a variation in the 2n number of the Columbidae species analyzed, ranging from 76 (*Z*. *auriculata*, *C*. *picui*, *C*. *passerina*, *P*. *cayennensis* and *C*. *talpacoti*) to 86 (*G*. *violacea* and *G*. *montana*) *L*. *verreauxi* and *C*. *livia* showed an intermediate 2n (78 and 80, respectively). Among the species, the karyotype of *G*. *violacea* was described for the first time, showing that this species has a karyotype very similar to another species of this genus, *G*. *montana*, both in terms of chromosome morphology and in the diploid number.

In birds, it is accepted that the presence of one pair of microchromosomes bearing 18S rDNA clusters is the ancestral state, considering that this is the condition observed in basal groups, such as Ratites and Galloanserae (Ladjali-Mohammedi *et al.*, 1999; Nishida-Umehara *et al.*, 2007), and also in many species belonging to more derived groups, such as some Passeriformes and Accipitriformes (Tagliarini *et al.*, 2011; dos Santos *et al.*, 2015). This characteristic seems to be conserved also in Columbiformes, since, with the exception of *Columbina picui*, which showed three pairs of microchromosomes bearing 18S rDNA clusters, the other eight species analyzed presented only one microchromosome pair bearing these clusters, including two other *Columbina* species. One of the most accepted causes of this variation, even among phylogenetically related species, is the transposition or translocation of these sequences (Nishida *et al.*, 2008; Kretschmer *et al.*, 2014).

Considering the microsatellite sequences, we applied eleven different oligonucleotide probes, which gave different results for each species, demonstrating that the analysis of these repetitive sequences may represent an important chromosome marker in evolutionary and phylogenetic studies in birds. Only one species, *C*. *picui*, did not show a signal for any of the sequences used. A possible explanation is that microsatellites have a characteristic mutational behavior, with rates that are 10 to 100,000 times higher than the average mutation rates in other parts of the genome (Gemayel *et al.*, 2010). Therefore, a microsatellite sequence can expand (addition of repeat units) or contract (deletion of repeat units) (López-Flores and Garrido-Ramos, 2012). It is possible that contraction of the microsatellites sequences occurred in *C*. *picui*, so the probes used were not complementary to the new sequence, considering the limitations inherent to FISH techniques, which needs at least 2–5 kb to be visible.

**Figura 6:**
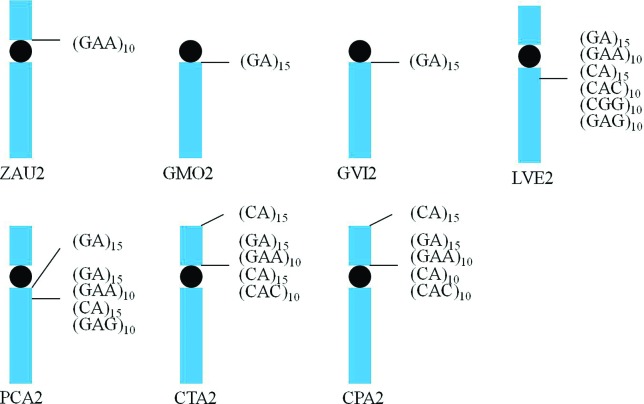
Distribution and localization of microsatellite sequences in chromosome 2 of seven Columbidae species: ZAU (*Zenaida auriculata*), LVE (*Leptotila verreauxi*), PCA (*Patagioenas cayennensis*), GVI (*Geotrygon violacea*), GMO (*Geotrygon montana*), CTA (*Columbina talpacoti*) and CPA (*Columbina passerina*).

Accumulation of microsatellites in pair 2 was observed in practically all species, (the exceptions were *C. livia* and *C. picui*), although in different positions (Figure 6), probably due to intrachromosomal rearrangements, such as inversions, which are very frequent among birds (Warren *et al.*, 2010; Kretschmer *et al.*, 2014, 2015; dos Santos 2015, 2017). Interestingly, while (GGA)_10_ produced signals in pair 2 of *Zenaida auriculata*, this sequence did not produce any signal in the two species of the genus *Geotrygon*. Instead, the sequence (GA)_15_ hybridized in pair 2 of *G. montana* and *G. violacea*. In the remaining species, a higher number of sequences accumulated in pair 2: *L*. *verreauxi* [(CA)_15_, (GA)_15_, (GAA)_10_, (CAC)_10_, (CGG)_10_ and (GAG)_10_]; *P*. *cayennensis* [(CA)_15_, (GA)_15_, (GAA)_10_ and (GAG)_10_]; *C*. *talpacoti* [(CA)_15_, (GA)_15_, (GAA)_10_ and (CAC)_10_], and; *C*. *passerina* [(CA)_15_, (GA)_15_, (GAA)_10_ and (CAC)_10_].

From a phylogenetic point of view, the occurrence of the same sequences found in the same position in pair 2 of different species could be a reflection of a common origin, as for example the sequences (CA)_15_, (GA)_15_, (GAA)_10_ and (CAC)_10_ in the species *L*. *verreauxi*, *C*. *talpacoti*, and *C*. *passerina*, and the three first ones in *P*. *cayennensis.* Furthermore, a more detailed analysis of these sequences in pair 2 of Columbidae species revealed that this pair is very informative about the karyotypical evolution in this group.

For instance, the presence of (GA)_15_ in pair 2 of *Geotrygon* species, which is telocentric in this species but submetacentric in most of the other ones, suggests the occurrence of a chromosomal rearrangement, such as an inversion or fission in this pair. However, if we consider that the 2n of *Geotrygon* is higher than that for the other species (2n=86), with pair 2 being slightly smaller (Figure 1), it seems that fission is the most probable rearrangement to have occurred in this genus. Moreover, the sequence (GA)_15_ hybridized in two different bands in the long arms of pair 2 in *P*. *cayennensis*, probably due to an inversion, which fragmented the block of repetitive sequences in two distinct ones. Similarly, the variation in the position of these repetitive sequences blocks in chromosome 2 – 2p in *C*. *passerina* and *C*. *talpacoti*, while 2q in *L*. *verreauxi*, *G*. *montana*, *G*. *violacea*, *P*. *cayennensis* – adds evidence for the occurrence of intrachromosomal rearrangements. A possible approach to test this hypothesis is the use of whole-chromosome probes of a species in which the syntenic group corresponding to GGA1 is found fragmented, such as *Leucopternis albicollis* (Falconiformes, Accipitridae), in which GGA2 corresponds to three different pairs (de Oliveira *et al.*, 2010).

The importance of repetitive sequences in chromosomal instability has been proposed by some authors (e.g. Ruiz-Herrera *et al.*, 2006). For example, the molecular characterization of evolutionary breakpoints in the genome of humans, primates and mouse has demonstrated that the genomic reorganizations mainly occur in regions with duplications or with some type of repetitive sequences, such as the dinucleotide (TA)n, or close to these regions (Kehrer-Sawatzki *et al.*, 2005; Fan *et al.*, 2002; Kehrer-Sawatzki *et al.*, 2002; Locke *et al.*, 2003). Although there is no single sequence responsible for the chromosomal instability, it is known that common fragile sites are enriched with A/T sequences and have the potential to form secondary structures (Schwartz *et al.*, 2006; Glover, 2006). These features may affect the DNA replication and lead to chromosomal instability (Ruiz-Herrera *et al.*, 2006). Interestingly, the dinucleotide (TA)_15_ did not produce any positive signals in our studies, revealing a possible characteristic intrinsic to the genome of birds. Although the absence of signals may reflect not only the inexistence of clusters of this sequence, it may instead represent a lower number of repetitions, considering the limitations inherent to FISH techniques, which needs at least 2–5 kb to be visible. This lower number of repetitions may be related to the small size of the genome of birds, at the expense of loss of repetitive sequences (Hughes and Hughes, 1995; Organ and Shedlock, 2009; Zhang and Edwards, 2012).

Concerning sex chromosomes, it is widely accepted that the accumulation of repetitive sequences plays an important role in the differentiation of the element found exclusively in the heterogametic sex – W or Y (Matsubara *et al.*, 2015). For instance, none of the sequences produced any signals in the Z chromosome, while different sequences were found accumulated in the W chromosome of the three species of which we analyzed female individuals: *C*. *livia* [(CAA)_10_, (CGG)_10_, (GA)_15_ and (GAG)_10_]; *G*. *violacea* [(GA)_15_ and (GAG)_10_], and *L*. *verreauxi* [(CA)_15_, (CAA)_10_, (CGG)_10_, (CAC)_10_, (GAG)_10_, (GAA)_10_ and (GA)_15_]. Of these, two were also found in the W chromosome in *Gallus gallus*: sequences (GA)_15_ and (GAG)_10_ (Matsubara *et al.*, 2015). Interestingly, these two sequences were shared by the three Columbidae species, possibly denoting some type of ancestral state. In fact, microsatellites are considered early colonizers of sex chromosomes and the differential accumulation of the same class of repeats on the W chromosome of distinct species reflects the inherent dynamism of these sequences (Charlesworth *et al.*, 2005).

In summary, this study demonstrated the ubiquitous presence of repetitive elements in the genome of several Columbidae species, highlighting their possible role in the chromosomal diversification within this group. In addition, our data reinforced the view that the existence of one pair of microchromosomes bearing 18S rDNA clusters is apparently an ancestral character retained in Columbidae, and that repetitive sequences did preferentially accumulate in the centromeric regions of macro and microchromosomes, as well as in the W chromosomes. Additionally, despite the fact that studies with repetitive sequences in birds are still incipient, the comparison of our data with the ones for Psittaciformes, Piciformes and Galliformes (Matsubara *et al.*, 2015; de Oliveira *et al.*, 2017; Furo *et al.*, 2017) shows interesting variation in accumulation sites for some of them, reinforcing microsatellites as important markers for studies on karyotype evolution.
